# Relationship between Periodontitis and Rheumatoid Arthritis: Review of the Literature

**DOI:** 10.1155/2015/259074

**Published:** 2015-08-04

**Authors:** Vilana Maria Adriano Araújo, Iracema Matos Melo, Vilma Lima

**Affiliations:** ^1^Departamento de Fisiologia e Farmacologia, Universidade Federal do Ceará (UFC), Rua Coronel Nunes de Melo, 1127 Rodolfo Teófilo, 60430-270 Fortaleza, CE, Brazil; ^2^Faculdade de Farmácia, Odontologia e Enfermagem, Universidade Federal do Ceará (UFC), Rua Alexandre Baraúna, 949 Rodolfo Teófilo, 60430-160 Fortaleza, CE, Brazil

## Abstract

Periodontitis (PD) and rheumatoid arthritis (RA) are immunoinflammatory diseases where leukocyte infiltration and inflammatory mediators induce alveolar bone loss, synovitis, and joint destruction, respectively. Thus, we reviewed the relationship between both diseases considering epidemiological aspects, mechanical periodontal treatment, inflammatory mediators, oral microbiota, and antibodies, using the keywords “periodontitis” and “rheumatoid arthritis” in PubMed database between January 2012 and March 2015, resulting in 162 articles. After critical reading based on titles and abstracts and following the inclusion and exclusion criteria, 26 articles were included. In the articles, women over 40 years old, smokers and nonsmokers, mainly constituted the analyzed groups. Eight studies broached the epidemiological relationship with PD and RA. Four trials demonstrated that the periodontal treatment influenced the severity of RA and periodontal clinical parameters. Nine studies were related with bacteria influence in the pathogenesis of RA and the presence of citrullinated proteins, autoantibodies, or rheumatoid factor in patients with PD and RA. Five studies investigated the presence of mediators of inflammation in PD and RA. In summary, the majority of the articles have confirmed that there is a correlation between PD and RA, since both disorders have characteristics in common and result from an imbalance in the immunoinflammatory response.

## 1. Introduction

Periodontitis (PD) is a chronic inflammatory disease where resident cells and preformed mediators induce leukocyte infiltration and progressive destruction of the tooth supporting tissues as a result of interaction between bacterial products, cell populations, and mediators in disease-susceptible individuals [1, 2]. This is also influenced by genetic and environmental risk factors and is characterized as a complex disease with multifactorial etiology [3, 4]. In this context, environmental factors, including oral hygiene/bacterial plaque, smoking, and stress, play an important role in the expression of PD [3]. Furthermore, it has been evidenced by some authors that there is a joint influence of polymorphisms in multiple genes [5], such as the genes of IL-10 [6] and IL-6 [7].

Polymorphonuclear neutrophils (PMNs) represent the first line of defense to protect the host from periodontal pathogens in the gingival sulcus and junctional epithelium. Data on the role of the pathogenesis of periodontitis are mixed. PMNs are a critical arm of defense against periodontitis, but bacterial evasion of the neutrophil microbicidal machinery coupled with delayed neutrophil apoptosis may transform the neutrophil from defender to perpetrator [8]. Actually, these cells can release a variety of factors, such as reactive oxygen species, collagenases, and other proteases, [1, 9], such as stimulation from a wide range of cytokines. In this scenario, macrophages can act as antigen-presenting cells, promoting the activation of lymphocytes [1]. Therefore, the cellular concentration of neutrophils in the inflammatory infiltrates decreases during the transition between gingivitis and periodontitis, in which there is a predominance of lymphocytes [9].

**Figure 1:**
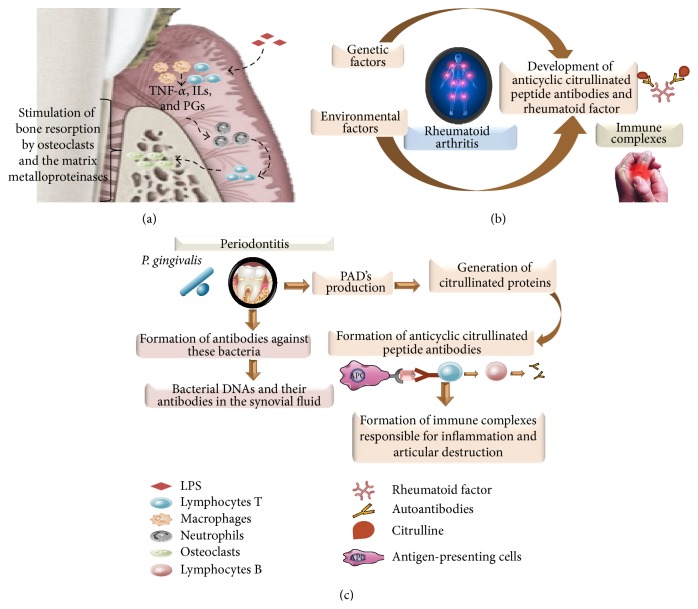
Scheme on the relationship between periodontitis and rheumatoid arthritis. (a) Pathogenesis of periodontitis and the effects promoted by lipopolysaccharides present in periodontopathogens. (b) The involvement of genetic and environmental factors in the development of rheumatoid arthritis. (c) Possible mechanisms that explain the relationship between rheumatoid arthritis and periodontitis.

It has been described that proinflammatory cytokines, prostaglandin E_2_, matrix metalloproteinase (MMP), nitric oxide (NO), and other inflammatory mediators play a crucial role in the pathogenesis of PD [10–12]. Moreover, an increase of TNF-*α*, IL-1*β*, IL-6, IL-11, and IL-17 can induce osteoclastogenesis by increasing the expression of Receptor Activator of NF-*κ*B Ligand (RANKL) and by reducing the osteoprotegerin (OPG) production in osteoblasts and stromal cells [13]. In fact, it was demonstrated that IL-17 and RANKL were overregulated and IL-10, an anti-inflammatory cytokine, and TGF-*β*1 were downregulated in active periodontal lesions compared with inactive lesions [14, 15] (Figure 1(a)).

Considering that an imbalance between bone formation and resorption is also linked to various diseases, studies suggest that PD may be a risk factor for other diseases [16], such as rheumatoid arthritis (RA) [17], but without consensus. Although pathogenesis of RA is not completely understood, it is recognized that the activation of the complement system is important in disease development [18], the abnormal response of circulating lymphocytes from patients, and an alteration in the structure of these cells, which contribute to the autoimmunity, immunosuppression, and the genesis of the disease [19]. Studies report there is a correlation between both PD and RA since the mechanisms for the development of RA have consonance with the pathogenesis of chronic PD. In fact, RA is defined as an inflammatory and autoimmune disease characterized by accumulation of leukocyte inflammatory infiltrate in the synovial membrane, as well as mediators such as PGE_2_, TNF-*α*, IL-1*β*, IL-6, IL-12, IL-17, IL-18, IL-33, granulocyte macrophage colony-stimulating factor (GM-CSF), Monocyte Colony-Stimulating Factor (M-CSF), RANKL, MMPs, and NO, all being found in the synovial fluid [20–24], and leading to synovitis and joint architecture destruction.

Some studies have suggested that the susceptibility of RA may be associated with genetic or environmental factors [25]. One of the most important genetic factors is the human leukocyte antigen (HLA) class II. Certain alleles of this antigen are often associated with the development of rheumatoid arthritis (HLA-DRB1^*∗*^0101, HLA-DRB1^*∗*^0102, HLA-DRB1^*∗*^0401, HLA-DRB1^*∗*^0404, HLA-DRB1^*∗*^0405, HLA-DRB1^*∗*^0408, HLA-DRB1^*∗*^0410, HLA-DRB1^*∗*^1001, and HLA-DRB1^*∗*^1402) [14]. Other factors include the allele of 620W of PTPN22 (protein tyrosine phosphatase nonreceptor type 22), a gene encoding tyrosine phosphatase that is involved in controlling the intracellular signaling triggered through T and B receptors [26]; C5-TRAF1, which can interfere with disease susceptibility and severity of the alteration in the structure, function, and levels of complement component c5/factor 1 associated with the TNF receptor [27]; gene encoding the CTLA4 (cytotoxic T lymphocyte antigen-4), the protein responsible for the regulation of T lymphocyte activation [28]; peptidylarginine deiminase (PAD2), the enzyme responsible for the generation of citrullinated proteins, which are related to the formation of anticyclic citrullinated peptide autoantibodies [29] (Figure 1(b)). With regard to environmental factors, smoking is a risk factor that duplicates the risk of developing RA, but its effect is limited to those with antibodies to citrullinated peptides [30, 31]. Other factors refer to the excessive consumption of coffee (more than 10 cups daily) which can be related to the development of the disease [32] and bacterial microbiota, including oral bacterial species which can participate in the etiopathogenesis of RA [33]. On the other hand, the intake of alcohol may exert a protective effect in rheumatoid arthritis in a dose-dependent manner [32]. The literature shows that the basic difference between both diseases is that RA is an inflammatory autoimmune disease, while PD is an immunoinflammatory disease of bacterial origin [9]. However, it is noteworthy that many epidemiological studies seem to dilute the subtle differences expressed by some parameters, though clinically important. Indeed, analyses of inflammatory mediators and other molecular markers are examples where the differences found in a trial with few participants could disappear in a large and diverse sample. In this sense, this review is a critical appraisal of studies that address potential associations of periodontitis with RA and with an overall comprehensive approach.

## 2. Methods

**Figure 2:**
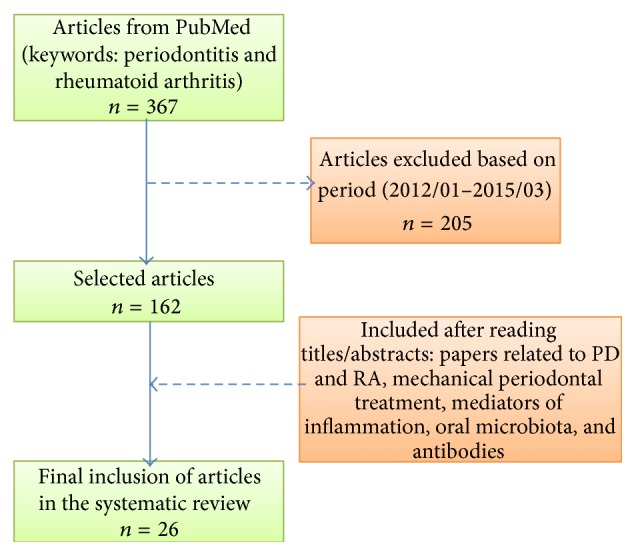
Search flow-chart and selection of articles for the review of the literature considering a bit more than the last three years. PD = periodontitis; RA = rheumatoid arthritis.

For this review, the US National Library of Medicine National Institutes of Health PubMed was searched by two independent researchers who agreed with the search criteria of studies with patients with both PD and RA and checked by a third researcher separately. The keywords* periodontitis* and* rheumatoid arthritis* were used and 367 articles published in English were found. The time period was limited from January 2012 to March 2015, and 162 references were found. Then, a critical reading based on titles and abstracts was made and 136 papers were excluded, such as reviews, assays* in vitro* and animal studies, articles that were not in English, studies not related to both PD and RA, case study, workshop, or unavailable and incomplete articles. Then, 26 articles were finally included for this review, which related to PD and RA, considering epidemiological aspects, mechanical periodontal treatment, mediators of inflammation, oral microbiota, and antibodies as seen in Figure 2.

## 3. Results

Table 1 shows demographic data, such as gender, age and habits, comorbidities and medications, and the relationship between both diseases investigated through clinical and epidemiological associations, presence of oral bacterial DNA in patients with RA, proinflammatory mediators, antibodies against bacteria, and autoantibodies, as well as the effects of mechanical periodontal treatment, related to the 26 selected articles.

In most articles (92.3%), the analyzed groups were mainly composed of women. Regarding age, most patients were 40 years old, except for the study of Dev et al. (2013) [45] and Ranade and Doiphode (2012) [37], whose patients were above 20 and 30 years old, respectively. Among the 26 articles, 57.7% [34–36, 38, 39, 42, 50–57, 59] used samples with smoker patients, while 30.8% established smoking as criteria for excluding [37, 41, 43, 45–49]. 11.5% did not mention smoking status of patients [40, 44, 58]. Comorbidities such as diabetes, Sjögren's syndrome, hypertension, cardiovascular disease, hyperlipidemia, renal disease, and osteoporosis/osteopenia have only been reported in studies of Mikuls et al. (2012) [35], Khantisopon et al. (2014) [54], and Gonzales et al. (2015) [59]. Regarding the medication used by patients, 50% [35–37, 40, 41, 44, 45, 49, 50, 52, 53, 55, 58] of the articles did not specify the pharmacological treatment. In the remainder of the studies, the most frequently reported treatment for rheumatoid arthritis included disease-modifying antirheumatic drugs (methotrexate, sulfasalazine, and leflunomide) [38, 39, 42, 43, 46, 51, 54, 56, 57, 59], biologic therapy (anti-TNF-*α*) [34, 38, 39, 42, 59], corticosteroids (prednisolone) [38, 42, 43, 46, 51, 54, 56, 59], and/or nonsteroidal anti-inflammatory drugs [43, 46–48, 51, 54, 57].

Among the selected trials, eight studies broached the epidemiological and clinical relationship of patients with PD and RA [38, 41, 44, 45, 49, 54, 56, 59], indicating a higher prevalence of PD in patients with RA, which have worse periodontal parameters. The effect of mechanical removal of foci of infection in the oral cavity on the severity of RA and periodontal clinical parameters were shown by four studies [37, 42, 46, 51], which demonstrated the beneficial effects of the mechanical treatment in the improvement of clinical parameters of RA. Two studies were related to the oral bacteria influence of the pathogenesis of RA [40, 52]. Seven trials highlighted the presence of citrullinated proteins and their antibodies, antibodies to* P. gingivalis* in patients with RA and periodontitis, and also the association between anti-*P. gingivalis* and periodontal parameters, and the titers of rheumatoid factor and antibodies anticyclic citrullinated peptide, which were also related to the severity of PD [35, 36, 39, 50, 53, 55, 57]. Regarding the inflammation in both diseases, five trials considered the mediators of inflammation to the PD and RA [34, 43, 47, 48, 58], such as MMP-9, TNF-*α*, IL-17, RANKL, and OPG. Considering the relationship between rheumatoid arthritis and periodontitis, only two articles showed no statistical significant association, while 24 studies have established this association, either by descriptive (3 studies) or statistical analysis (21 studies).

## 4. Discussion

In this review, demographic data and other aspects that can modify one or both diseases were presented, as well as the relationship between both diseases investigated through clinical and epidemiological associations, effects of mechanical periodontal treatment, presence of oral bacterial DNA in patients with RA, proinflammatory mediators, antibodies against bacteria, and autoantibodies.

The articles showed higher prevalence of female patients. This aspect was interesting, as a possible relationship between female sex hormones and susceptibility of rheumatoid arthritis had been reported in the literature, so that low levels of those hormones at menopause promote the risk of developing the disease early [60]. However, a protective role of oral contraceptives on the risk for rheumatoid arthritis in women is still controversial [61–63]. On the other hand, there is strong evidence that estrogen deficiency influences the severity of periodontitis, since worse periodontal parameters were observed as bleeding on probing, gingival recession, and clinical attachment loss in postmenopausal women with osteoporosis [64].

The smoking status was also recorded in the selected studies. Cigarette smoking is considered an important risk factor for the development of rheumatoid arthritis, since it was demonstrated that lifelong cigarette smoking was positively associated with the risk of RA even among smokers with a low lifelong exposure [65]. Moreover, it has been related that smoking interacts with HLA-DR SE genes and increases the risk of anti-CCP antibodies in patients with rheumatoid arthritis [66]. Regarding the periodontium, it was shown that smokers presented greater probing depths, when compared to the probing depths of patients who never smoked [67].

The literature shows that PD does not usually require pharmacological treatment, except for mechanical periodontal treatment as routine. In this review, this fact was also observed, while half of the studies had shown that rheumatoid arthritis involved some pharmacological approach. The use of disease-modifying antirheumatic drugs (DMARDs) aims to reverse the symptoms of the disease, reduce the progression of joint damage, and consequently improve the quality of life of patients [68]. The conventional synthetic DMARDs include methotrexate, sulfasalazine, and leflunomide; the available tumor necrosis factor inhibitors (adalimumab, etanercept, and infliximab), the T cell costimulation inhibitor (abatacept), the anti-B cell agent (rituximab), and the interleukin-6 receptor blocking monoclonal antibody are included in biological DMARDs [69]. These medications may be associated with glucocorticoids (GC) or nonsteroidal anti-inflammatory drugs (NSAIDs). The long-term, low-dose glucocorticoid and NSAIDs therapy were shown to reduce joint symptoms, pain, and other systemic manifestations [70, 71]. Although these benefits are present, the long-time treatment with GC and methotrexate decreased immune response and promoted oral changes, such as candidiasis, periodontitis, and oral ulceration besides impaired saliva secretion [72]. Indeed the literature demonstrated that patients on corticosteroids exhibited higher levels of candidiasis, clinical attachment loss, and probing pocket depth [73]. These aspects, at least in part, may contribute to the worse periodontal status of RA patients when compared to healthy patients. Moreover, the use of medications referred to in half of the articles could compromise the evaluation of this review. However, it is noteworthy that the other half of the articles did not use any medication [35–37, 40, 41, 44, 45, 49, 50, 52, 53, 55, 58].

Analysing the articles, it was observed that most patients with RA showed a significant increase in the incidence of PD as compared to healthy individuals, while only few articles concluded the opposite, probably due to the lack of standardization of parameters in evaluating the different types of periodontitis. Although epidemiological studies outlined by Dev et al. (2013) [45] have not observed a significant RA incidence in subjects with periodontitis where these authors suggested that periodontitis is an independent factor for RA, several other studies have shown that patients with RA were more susceptible to the development of periodontitis [38, 44], since these patients had worse periodontal parameters, such as clinical attachment level [37, 56], alveolar bone loss [56, 59], probing depth [37, 49], plaque index, and bleeding on probing [37, 41, 54]. Indeed, the mechanical periodontal treatment as scaling and root planning in the control of periodontal infection interfered not only with the severity of RA but also with the periodontal clinical parameters [74]. This result can be explained by a reduction in the foci of oral bacteria, and therefore the low levels of inflammation demonstrated a decrease of DAS28 (disease activity score in 28 joints) and serum levels of IL-1*β*, TNF-*α*, C-reactive protein, and erythrocyte rate sedimentation [37, 42, 46, 51]. In this sense, studies have defended the hypothesis that oral infections play an important role in the pathogenesis of RA, promoting the citrullination of proteins, which can be based on the detection of bacterial DNA using the techniques of DNA isolation (PCR and DNA-DNA hybridization) and high titers of antibodies against bacteria in synovial fluid and serum samples from patients with RA [40, 51, 52]. Most of the studies have shown the presence of oral bacteria in patients with RA, highlighting* P. gingivalis *and* F. nucleatum* [40, 52]. Markedly,* P. gingivalis* is the most elucidated in the development of RA, and studies using animal models have demonstrated the potential of this proinflammatory bacterium promoting the development of experimental arthritis and increased serum levels of C-reactive protein, TNF-*α*, IL-1*β*, IL-17, MMP-13, and RANKL [75]. Furthermore, RA is an autoimmune disease characterized by autoantibodies specific for citrullinated peptide antigen (anticyclic citrullinated peptide), which are synthetized by peptidylarginine deiminase and characterized as the most specific markers for the diagnosis of the disease [76, 77]. Considering that the* P. gingivalis* is regarded as being capable of expressing this enzyme (PAD), it is suggested that infection with this microorganism could influence the pathogenesis of RA [78, 79]. These citrullinated proteins were also found in periodontal tissues, indicating a link between these peptides generated in the oral cavity and those observed in articular tissues [36, 80].

Additionally, the presence of antibodies to* P. gingivalis* was investigated. Although Seror et al. (2015) [57] have not detected this, antibody titres significantly differ between early rheumatoid arthritis and healthy controls. Other studies observed the antibodies to* P. gingivalis* in patients with RA and severe periodontitis [39] and were associated with probing depth and clinical attachment level and the titers of rheumatoid factor and anticyclic citrullinated peptide autoantibodies [35, 50], which may be found in patients with RA and related to the severity of periodontitis [55]. In summary, the studies suggested that* P. gingivalis* might play a role in the pathogenesis of RA. The response in periodontitis was related to uncitrullinated peptide, suggesting that these peptides break tolerance and can be involved in pathogenesis of RA [53] (Figure 1(c)).

Inflammatory conditions and mechanisms for bone destruction in PD and RA have many similarities. Most of the studies have found high levels of proinflammatory cytokines and other mediators of inflammation, such as MMP-9 [58], TNF-*α* [48], IL-17, RANKL, and OPG [47]. Moreover, it was demonstrated that the hypomethylated status, a single region of the IL-6, may contribute to elevated serum levels of this cytokine, implying a role in the pathogenesis of these diseases [34], while the anti-inflammatory cytokines in the GCF, such as IL-4 and IL-10, showed no consensus among studies regarding the differences observed among individuals with PD and RA [43]. In addition, hypotheses have been proposed to explain the relationship between periodontitis and systemic diseases, such as rheumatoid arthritis. In the literature, studies have suggested that chronic periodontitis generates local constant high levels of microparticles, which have been considered inflammatory biomarkers or mediators responsible for distant cell signalling and regulation [81]. Moreover, it has been reported that these microparticles play an important role in thrombosis and angiogenesis and mediate cellular communication by transferring mRNAs and microRNAs from the cell of origin to target cells [82]. Thus, the microparticle participation and its spread into the bloodstream could constitute the explanation to the increased risk for systemic disease in patients with periodontitis [83].

Despite these evidences showing a link between rheumatoid arthritis and periodontitis, the exact mechanisms involving this association have not been fully elucidated. Thus, well-designed longitudinal multicentre clinical trials and further studies with sufficient sample sizes are required to determine the biochemical processes and clinical relationships between these chronic inflammatory conditions. Moreover, these studies should consider other potential confound factors such as the drugs administered for the treatment for each disease or differences in oral hygiene or smoking habits in these patients.

## 5. Conclusion

The majority of the articles have confirmed that there is a correlation between PD and RA, since both disorders have characteristics in common and result from an imbalance in the immunoinflammatory response. Although it is necessary to highlight the importance of the mechanical treatment for periodontitis and pharmacological treatments mainly for RA patients, more research is needed to assess whether the coexistence of both diseases can affect the clinical signs of periodontitis and systemic markers of rheumatoid arthritis and strengthen the capacity of oral bacteria to stimulate an autoimmune response, thus establishing that cell constituents or mediators could share common pathophysiological pathways for both diseases and therefore define the best therapy.

## Figures and Tables

**Table 1:** Summary of papers relating to the relationship between periodontitis and rheumatoid arthritis.

Authors	Study population	Demographic characteristics	Exclusion criteria	Periodontal disease evaluation	RA treatment	Results	Association of PD × RA
Ishida et al., 2012 [34]	30 RA patients; 30 patients with PD; 30 healthy volunteers	RA (27 females and 3 males; 66 ± 2 years; 3 smokers); PD (20 females and 10 males; 62.3 ± 1.5 years; 1 smoker and 2 former smokers); healthy (20 females and 10 males; 53.4 ± 2.7 years; nonsmokers)	Periodontal therapy 6 months prior to examination Less than 15 teeth Diabetes mellitus and pregnancy	CAL, PPD, and missing teeth	Anti-TNF-*α*	The hypomethylated status, a single region of the IL-6, may contribute to elevated serum levels of this cytokine, implying a role in the pathogenesis of PD and RA	*P* < 0.0001

Mikuls et al., 2012 [35]	Patients: 171 autoantibody negative; 75 autoantibody positive; 38 high risk based on the presence of a positive ACPA or positivity to 2 or more RF assays	Negative antibody (69% female; 44 ± 14 years; 37% smokers; 5% with DM); positive antibody (73% female; 48 ± 15 years; 31% smokers; 4% with DM); high risk (76% female; 51 ± 16 years; 29% smokers; 5% with DM)	Age < 18 years	Not analyzed	Not informed	Anti-*P. gingivalis* concentrations were higher in high-risk, with autoantibody (anti-CCP and RF), positive group than in the autoantibody negative group	*P* = 0.01

Nesse et al., 2012 [36]	15 patients with PD; 6 healthy volunteers; 4 RA patients	PD and Ab5612+ (63% female; 48.9 ± 11.4 years; 50% smokers); PD and Ab5612− (71% female; 53.6 ± 17.9 years; 29% smokers); PD and F95+ (50% female; 55 ± 12.4 years; 40% smokers); PD and F95− (100% female; 43.2 ± 16.3 years; 40% smokers); PD either+ (58% female; 52.3 ± 14 years; 42% smokers); PD both− (100% female; 46.2 ± 18.5 years; 33% smokers); controls either+ (100% female; age: 28.5 ± 5.5 years; 50% smokers); controls either− (50% female; 28 ± 8.6 years; 25% smokers)	Other systemic conditions (excluding RA)	PPD	Not informed	Formation of citrullinated proteins in periodontal tissues was shown, which appear to be a variety similar to those observed in RA synovial tissue affected by RA	—

Ranade and Doiphode, 2012 [37]	40 RA patients; 40 healthy volunteers	80% female 20–70 years	Systemic diseases; medications that affect the periodontium; tobacco habit Dental treatment (a month before)	ABL, CAL, PPD, GI, and PI	Not informed	High prevalence of mild to moderate PD in patients with RA presenting significantly higher GI, PI, PPD, and CAL, when compared to healthy volunteers	*P* < 0.001

Scher et al., 2012 [38]	31 patients with new-onset RA; 34 chronic RA patients; 18 healthy volunteers	New-onset RA (68% female; 42.2 years; 16% smokers, 16% former smokers, and 68% nonsmokers); chronic RA (79% female; 47.7 years; 6% smokers; 24 former smokers; 70% nonsmokers); healthy (65% female; age: 42.2 years; 6% smokers; 16% former smokers; 78% nonsmokers)	Recent use of any antibiotic therapy; current extreme diet; inflammatory bowel disease Malignancy; consumption of probiotics; tract surgery leaving permanent residua; liver, renal, or peptic ulcer diseases	CAL, PPD, and BOP	Corticoids, DMARDs, and biologic therapy	New-onset RA patients exhibit a high prevalence of PD at disease onset; the colonization with *P. gingivalis* correlates with PD severity; overall exposure was similar among groups	*P* < 0.01

Smit et al., 2012 [39]	95 RA patients; 44 non-RA controls; 36 healthy volunteers	RA (68% female; 56 ± 11 years; 23% current smokers; 40% former smokers); non-RA controls (57% female; 54 ± 9.7 years; 27% current smokers; 43% former smokers); healthy (56% female; 34 ± 15 years; 14% current smokers)	Age < 18 years; edentulism; diabetes mellitus; active thyroid disease; nonoral infections Malignancy; myocardial infarction or stroke; pregnancy Antibiotic use	BOP, PPD, and CAL	DMARDs and anti-TNF-*α*	Association between PD and RA and the increased prevalence of PD in patients with RA	*P* < 0.001
						Anti-*P. gingivalis* titers were higher in RA patients with severe PD compared with non-RA patients	*P* < 0.05

Témoin et al., 2012 [40]	11 RA patients; 25 patients with OA	RA (100% female; 45–70 years); OA (9 males and 16 females; 50–80 years)	Antibiotic use Edentulism	Not analyzed	Not informed	Bacterial DNA was detected in 13.9% of RA patients; *F. nucleatum* consisted in the pathogen most prevalent	—

Torkzaban et al., 2012 [41]	53 RA patients; 53 healthy volunteers	RA (41.5 years); healthy (43.5 years); 58 females and 48 males	<7 teeth; systemic diseases such as diabetes or Sjögren's disease; antibiotics use; treatment for PD; immunosuppressive drugs; smokers	PI, BOP, and CAL	Not informed	Patients with RA had a higher percentage of sites presenting plaque, BOP, and CAL	*P* < 0.001

Bıyıkoğlu et al., 2013 [42]	10 patients with PD and RA; 15 patients with PD	PD and RA (9 females; 46.6 ± 8 years; 8 smokers); PD (6 females; 46.73 ± 7 years; 9 smokers)	Systemic disease or infection other than RA; history of antibiotic therapy Periodontal treatment <10 teeth	PPD, CAL, BOP, and PI	MTX, leflunomide, prednisolone, chloroquine, sulfasalazine, anti-CD20, and anti-TNF-*α*	The nonsurgical periodontal treatment reduced the clinical periodontal parameters and promoted an improvement in the scores of RA	*P* < 0.001

Cetinkaya et al., 2013 [43]	17 RA patients; 16 patients with PD; 16 healthy volunteers	RA (14 females and 3 males; 47.82 years) PD (6 females and 10 males; 44 years); healthy (8 females and 8 males; 28 years)	Conservative or prosthetic restorations; caries at the anterior region; systemic or local disease with an influence on the immune system (cancer and cardiovascular and respiratory diseases); history of hepatitis or HIV infection; immunosuppressive chemotherapy; current pregnancy or lactation; antibiotic prophylaxis; history of antibiotic therapy Periodontal treatment <18 years; smokers	PI, GI, PPD, and CAL	MTX, sulfasalazine, leflunomide, NSAIDs, and corticoids	No significant differences in the levels of pro- and anticytokine between PD and RA were observed	*P* > 0.05

Chen et al., 2013 [44]	13779 RA patients; 137790 non-RA patients	RA (77.4% female; 52.6 ± 14.4 years); controls (77.4% female; 52.4 ± 15.4 years); comorbidities: diabetes mellitus and Sjögren's syndrome	Age < 16 years	Periodontal surgery, number of PD-related visits	Not informed	PD severity was related to a history of periodontal surgery, more PD-related visits, and higher costs of medical care; an association between periodontitis and incident RA was demonstrated	*P* < 0.001

Dev et al., 2013 [45]	852 patients with PD; 668 healthy volunteers	52.8% female and 47.2% male 30–70 years	Smokers; diabetes mellitus; periodontal therapy (3 months before); antibiotic use (3 months before); systemic disease and osteoporosis; antibiotic prophylaxis; pregnancy; lactation	PPD, BOP, and CAL	Not informed	Moderate to severe periodontitis is an independent risk factor for RA	—

Erciyas et al., 2013 [46]	30 RA patients with moderate to high disease activity and chronic PD (LDA); 30 RA patients with low disease activity and chronic PD (MHDA)	LDA (25 females; 42.6 ± 10.05 years); MHDA (22 females; 43.83 ± 10.97 years)	Periodontal therapy (6 months); presence of any other systemic diseases; smokers; <18 teeth Antibiotic therapy	PI, PPD, CAL, and BOP	DMARDs Corticoids NSAIDs or anti-TNF-*α*	SRP might prove beneficial in reducing RA severity as measured by ESR, CRP, TNF-*α* levels in serum, and DAS28 in RA patients with chronic periodontitis	*P* < 0.001 *P* < 0.05 related to TNF-*α*

Gümüş et al., 2013 [47]	17 RA patients; 19 patients with OPR; 13 healthy volunteers	RA (17 females; 44 years) OPR (19 females; 58 years) Healthy (13 females; 54 years)	Systemic disease; antibiotic use (6 months); corticosteroids; *β*-blockers use; diabetes mellitus; periodontal therapy (6 months) <10 teeth; smokers	PPD, CAL, and BOP	NSAIDs	Concentrations in serum and GCF of RANKL and OPG were significantly higher and lower, respectively, in patients with RA when compared to individuals with OPR and healthy volunteers; the total counts of the IL-17 and IL-17F were significantly higher in patients with RA compared to the control group	*P* < 0.05

Gümüş et al., 2013 [48]	17 RA patients; 19 patients with OPR; 13 healthy volunteers	RA (17 females; 44 years) OPR (19 females; 58 years) Healthy (13 females; 54 years)	Systemic disease Antibiotic use (6 months); corticosteroids; *β*-blocker use; diabetes mellitus; periodontal therapy (6 months); <10 teeth; smokers	PPD, CAL, BOP, and PI	NSAIDs	Despite the long-term use of various anti-inflammatory drugs in RA and osteoporosis, patients involved in this study showed an increase in gingival crevicular and serum levels of TNF-*α*	*P* < 0.05

Joseph et al., 2013 [49]	100 RA patients; 112 healthy volunteers	RA (76 females and 24 males; 46.54 ± 8.5 years) Healthy (86 females and 26 males; 45.91 ± 9.76 years)	Systemic diseases; smokers; conditions that may alter the serum CRP and blood ESR levels; antibiotic use; periodontal therapy	GI, PPD, CAL, missing teeth, and OHI-S	Not informed	Patients with RA, compared to healthy volunteers, showed a significant difference in PPD and CAL, and 58% of patients with RA had moderate to severe PD	*P* < 0.05

Lappin et al., 2013 [50]	38 RA patients; 36 healthy volunteers	RA (17 females and 21 males; 31–70 years; 24 nonsmokers and 16 smokers); healthy (16 females and 20 males; 30–65 years; 20 nonsmokers and 16 smokers)	Systemic disease; previous antibiotic use (3 months)	PPD, CAL, BOP, and missing teeth	Not informed	Although smokers have shown lower antibody titers, individuals with periodontitis showed higher levels of anti-CCP antibodies	*P* < 0.001

Okada et al., 2013 [51]	55 RA patients	Treatment group (84.6% female; 60.7 years; 9 former smokers and 17 nonsmokers); control group (82.8% female; 62.7 years; 11 former smokers and 18 nonsmokers)	Diabetes mellitus; pregnancy; antibiotic use (3 months); periodontal therapy (3 months)	GI, PI, CAL, BOP, and PPD	Corticoids, DMARDs, and NSAIDs	SRP decreased RA parameters and serum levels of IgG to *P. gingivalis*	*P* < 0.01
						Citrulline in patients with RA	*P* < 0.04

Reichert et al., 2013 [52]	42 RA patients; 114 healthy volunteers	Healthy (40.4% female; 53.8 ± 16.7 years; 10.7% smokers, 14.3% former smokers, and 75% nonsmokers); RA (52.4% female; 56.1 ± 15.2 years; 14.3% smokers, 11.9% former smokers, and 73.8% nonsmokers)	Pregnancy; antibiotic use; periodontal therapy	BOP, CAL, and PI	Not informed	There was a significant amount of *P. gingivalis* DNA in synovial fluid and in subgingival plaque from patients with RA	*P* < 0.05

De Pablo et al., 2014 [53]	96 patients with PD; 98 without PD	PD (62% female; 46 ± 8.9 years; 24% smokers) Without PD (59% female; 29 ± 7.3 years; 22% smokers)	Pregnancy; lactation; antibiotic and NSAIDs use (3 months before); vitamin supplementation (3 months before); regular mouthwash; dietary requirements (celiac disease)	Not analyzed	Not informed	Serum antibodies were significantly higher in patients with PD compared with those without PD for antibodies against CEP-1, REP-1, vimentin, and fibrinogen	*P* < 0.0001
						Cit-vim	*P* = 0.003

Khantisopon et al., 2014 [54]	196 RA patients	87% female; 51.7 ± 9.7 years; 78% nonsmokers, 30.69% with hypertension; 34.16% with dyslipidemia; 2.97% with DM; 2.47% with chronic kidney disease; 58.97% with osteoporosis; and 23.08% with osteopenia.	Pregnancy; lactation; systemic conditions that could affect the progression of periodontal disease, such as uncontrolled diabetes mellitus, severe hypertension, severe renal insufficiency, or malignancies; antibiotic uses	GI, PI, CAL, PPD, and gingival recession	MTX, prednisolone, DMARDs, and diclofenac	RA Patients had a high prevalence of moderate or severe periodontitis	—
						Increasing age, the male sex, history of previous or current smoking, and high PI were associated with the severity of periodontal disease	*P* < 0.02

Mikuls et al., 2014 [55]	287 RA patients; 330 healthy volunteers	RA (63% male; 59 years; 19% smokers, 43% former smokers; 38% nonsmokers); healthy (60% male; 59 years; 11% smokers, 35% former smokers, and 54% nonsmokers)	Tetracycline or antibiotic use (6 months); cyclosporine or dilantin; antibiotic prophylaxis prior to dental probing	PPD, BOP, PI, and gingival recession	Not informed	Periodontitis was more common in patients with RA positive for anticyclic citrullinated peptide; there was an association between periodontitis and the number of inflamed joints and RF Antibodies specific for anticyclic citrullinated peptide were higher in patients with *P. gingivalis* subgingival plaque	*P* < 0.02

Wolff et al., 2014 [56]	22 RA patients; 22 healthy volunteers	68% female; 51.7 ± 9.7; 14% smokers	Current therapy with biological DMARDs; poor oral hygiene or disabilities that interfere with adequate oral hygiene; periodontitis as a manifestation of systemic disease; periodontal therapy within the past 5 years; professional to antibiotics use; pregnancy or nursing during the past 6 months	PPD, BOP, GI, PI, and CAL	DMARDs and corticoids	PPD, BOP, and CAL were increased in RA patients when compared to healthy volunteers	*P* < 0.001

Seror et al., 2015 [57]	694 early-RA patients; 79 healthy controls; 61 patients with PD; 54 patients with sicca	RA (78.2% female; 48.5 ± 12.3 years; 48% ever smokers); healthy (84.6% female; 47.6 ± 11.9 years; 16.2% ever smokers); sicca (85.2% female; 48.9 ± 11.5 years; 37.3% ever smokers); PD (41% female; 50.7 ± 8.3 years; 65.6% ever smokers)	DMARDs (except within the 15 days before inclusion) or steroids use; inflammatory rheumatic disease other than RA	Not analyzed	NSAIDs and DMARDs	Anti-*P. gingivalis* antibody titres did not significantly differ between early-RA patients and healthy controls, sicca controls, or PD controls	*P* = 0.66 *P* = 0.53 *P* = 0.17

Silosi et al., 2015 [58]	21 healthy controls, 16 with active RA, 14 with PC, and 12 RA-CP association	Controls (7 males and 14 females; 35–58 years) RA (4 males and 12 females; 38–62 years); PC (6 males and 8 females; 39–68 years) RA-PC (3 males and 9 females; 38–62 years)	History of medication other than NSAIDs Drugs (6 months); periodontal treatment; pregnancy; hormonal or vitamin therapy	PI, BOP, and PPD	Not informed	Differences of serum MMP-9 between RA and CP groups and control	*P* < 0.01
						Serum levels of MMP-9 were similar in RA and RA-CP	*P* > 0.05
						Increased MMP-9 CGF levels in RA-CP subjects as compared to CP	*P* < 0.05

Gonzales et al., 2015 [59]	287 with RA and 330 controls with OA	RA (63% male; 59 ± 12 years; 38% never smokers; 43% former smokers; 19% current smokers; 18% with DM; 45% hypertension; 13% cardiovascular disease; 11% osteoporosis); OA (60% male; 59 ± 11 years; 54% never smokers; 35% former smokers; 11% current smokers; 25% with DM; 57% hypertension; 10% cardiovascular disease; 15% osteoporosis)	Tetracycline or related antibiotic use (6 months); antibiotic premedication Pregnancy or breastfeeding; prior use of cyclosporine or phenytoin; systemic inflammatory disease	ABL	MTX, prednisolone, and biologic therapy	ACPA-positive patients with RA had a statistically significantly higher mean percentage of sites with ABL >20% than patients with OA	*P* = 0.03
						After multivariate adjustment, greater ABL was significantly associated with higher serum ACPA concentration,	*P* = 0.004
						DAS28, health assessment questionnaire disability,	*P* = 0.023
						tender joint count,	*P* = 0.05
						and joint space	*P* = 0.02
						narrowing scores among patients with RA	*P* = 0.05

ABL, alveolar bone loss; anti-CCP, anticyclic citrullinated peptide; anti-TNF-*α*, tumor necrosis factor-alpha antagonists; BI, bleeding index; BOP, bleeding on probing; CAL, clinical attachment level; CI, calculus index; DAS28, disease activity score in 28 joints; DM, diabetes mellitus; DMARDs, disease-modifying antirheumatic drugs; ESR, erythrocyte sedimentation rate; GAP, generalized aggressive periodontitis; GBI, gingival bleeding index; GBTI, gingival bleeding time index; GCF, gingival crevicular fluid; GI, gingival index; HCQ, hydroxychloroquine; IL, interleukin; JIA, juvenile idiopathic arthritis; LAP, localized aggressive periodontitis; MMP, matrix metalloproteinase; MTX, methotrexate; NSAIDs, nonsteroidal anti-inflammatory drugs; OA, osteoarthritis; OHI-S, oral hygiene index-simplified; OPR, osteoporosis; PBI, papillary bleeding index; PD, periodontitis; PI, plaque index; PPD, probing pockets depths; PsA, psoriatic arthritis; PSI, periodontal screening index; RA, rheumatoid arthritis; RANK, Receptor Activator of Nuclear Factor *κ*B; RANKL, Receptor Activator of Nuclear Factor *κ*B Ligand; RF, rheumatoid factor; SRP, scaling and root planning; TNF-*α*, tumor necrosis factor alpha; VPI, visible plaque index. *P* < 0.05 was considered significant.
